# The Antibacterial Activity of Erythrocytes From Goose *(Anser domesticus)* Can Be Associated With Phagocytosis and Respiratory Burst Generation

**DOI:** 10.3389/fimmu.2021.766970

**Published:** 2022-01-13

**Authors:** Youcheng Yang, Jiajun Chen, Linqing Lu, Zizheng Xu, Feng Li, Minxuan Yang, Jun Li, Li Lin, Zhendong Qin

**Affiliations:** ^1^ Guangdong Provincial Water Environment and Aquatic Products Security Engineering Technology Research Center, Guangdong Province Key Laboratory of Waterfowl Healthy Breeding, College of Animal Sciences and Technology, Zhongkai University of Agriculture and Engineering, Guangzhou, China; ^2^ School of Science and Medicine, Lake Superior State University, Sault Ste. Marie, MI, United States

**Keywords:** red blood cells (RBCs), *Anser domesticus*, respiratory burst, phagocytosis, antibacterial activity

## Abstract

In the lumen of blood vessels, there are large numbers of erythrocytes, which are approximately 95% of the total blood cells. Although the function of erythrocytes is to transport oxygen in the organism, recent studies have shown that mammalian and teleost erythrocytes are involved in the immune response against bacterial infections. However, the immune mechanisms used by avian erythrocytes are not yet clear. Here, we demonstrated that erythrocytes from goose have the ability to phagocytose as well as conduct antimicrobial activity. Firstly, we revealed the phagocytosis or adhesion activity of goose erythrocytes for latex beads 0.1-1.0 μm in diameter by fluorescence microscopy, and scanning and transmission electron microscopy. The low cytometry results also proved that goose erythrocytes had a wide range of phagocytic or adhesion activity for different bacteria. Followed, the low cytometry analysis data further explored that the goose erythrocytes contain the ability to produce reactive oxygen species (ROS) and inducible nitric oxide synthase (iNOS) in response to bacterial stimulation, and also up-regulated the expression of NOX family includes NOX1 and NOX5. Finally, we also found that goose erythrocytes showed a powerful antibacterial activity against all the three bacteria, meanwhile the stimulation of three kinds of bacteria up-regulated the expression of inflammatory factors, and increased the production of antioxidant enzymes to protect the cells from oxidative damage. Herein, our results demonstrate that goose Erythrocytes possess a certain phagocytic capacity and antioxidant system, and that the antimicrobial activity of erythrocytes can occurred through the production of unique respiratory burst against foreign pathogenic bacteria, which provides new clues to the interaction between bacteria and avian erythrocytes.

## Introduction

As an herbivorous waterfowl, goose meat and eggs are rich in nutritional value and have a unique taste. Goose is reared in Central Europe and Asia due to the economic importance of their meat, eggs, and down feathers (1, 2). The annual global consumption of goose meat is approximately 2.23 million tons (3). Goose have a significant role in the agricultural economy, and China is the world’s largest goose breeding country, with approximately 93% of the world’s goose production (4). In recent years, as human demand for eggs and meat are increased, and the scale and density of global livestock are also increased rapidly. Accordingly, a series of bacterial are also increased, and the environmental pollution caused by high density of goose, which all compromise animal health and immune response, already caused severe economic losses (5). Previous studies have shown that the main bacterial pathogens of poultry include avian pathogenic *Escherichia coli*, *Pasteurella multocida*, Salmonella, and *Staphylococcus aureus*, and these are very dangerous pathogens because they greatly limit the healthy development of poultry (6). Therefore, it is of great research significance to study the mechanisms of prevention and control of bacterial infectious diseases in goose populations.

Erythrocytes are well-known for their major function in exchange of gases during respiration. However, recent studies revealed that erythrocytes are also involved in the immune regulation of the organism, and can immunologically adhere to and kill invading pathogens (7–9). Compared to mammals, non-mammalian erythrocytes do possess a nucleus and organelles, and these enable complex cellular processes such as gene expression and protein synthesis to be carried out (10). Previous studies have determined that mammalian erythrocytes have a role in regulating inflammatory factors, such as tumor necrosis factor-α (TNF-α) and interleukin-1β (IL-1β) in the presence of lipopolysaccharide (LPS) (11). After LPS or bacterial stress, the hemoglobin of human erythrocytes releases free radicals that trigger the production of antibacterial reactive oxygen species (ROS), which kill pathogens by disrupting their cell walls and cell membranes (12, 13). It has been reported that the mammalian erythrocytes have a regulatory role in the innate immune response, and CD71^+^ erythrocytes with immunosuppressive functions are found in humans and mice, these erythrocytes have an immunosuppressive function *via* arginase-2 in mice (14). Recent, the study has been identified and reported that rainbow trout erythrocytes can respond to fungal infection of the host with a corresponding immune response, and also can phagocytose *Candida albicans* as well as present it to macrophages (15). In addition, it has been reported that the erythrocytes of grass carp (*Ctenophyngodon idella*) were capable of phagocytosis and were antibacterial because they could kill bacteria through their production of ROS (16). In avian, the previous study revealed that the macrophages and platelets play a co-clearance role in preventing *S. aureus* further spread in the organism (17). Previous study reported that the chicken erythrocyte transcription factor nuclear factor κB (NF-κB) gene plays an important role in the inflammatory response after low pathogenic avian influenza virus (LPAIV H9N2) infection, which indicates that chicken erythrocytes are involved in the organism’s immune response to LPAIV H9N2 (18). Meanwhile, chicken erythrocytes were also found to be involved in the organism’s immune response against the virus after chickens were infected with Marek’s disease virus (MDV) (19). However, there is still much less information on the potential antimicrobial mechanisms of avian erythrocytes. It is well-known, the ROS plays a vital role in antibacterial response, which was regulated by NOX family, includes NOX1-5, DUOX1, and DUOX2. And NADPH oxidases are composed of various subunits including cytoplasmic proteins (p47phox and p67phox) and membrane proteins (p91phox and p22phox). Activated cytoplasmic proteins can form complexes with membrane proteins within the cell membrane to generate ROS that kill pathogens (16).

In this study, we selected goose erythrocytes as an experimental model to study the functional role and potential antibacterial mechanisms of avian erythrocytes. To accomplish this goal, flow cytometry, fluorescence microscopy, scanning electron microscopy, transmission electron microscopy and so on were used to explore the role of goose erythrocytes and its antimicrobial mechanisms against antigens. Our results provide a new insight into the immune mechanisms of goose erythrocytes, and pave new strategic approaches for effective control of goose diseases.

## Materials and Methods

### Experimental Animals and Reagents

Magang goose (*Anser domesticus*) were purchased from a farm in Guangdong Province, China, and 12-week-old goose were used in this experiment. The goose was maintained under natural light and temperature conditions for at least 2 weeks and fed commercial diets according to the recommended nutritional standard for the breed. Food and water were sterilized before being fed to the goose. Latex beads of different diameters (0.5 μm, 0.8 μm, 1.0 μm, and 2.0 μm) were purchased from Sigma-Aldrich. *S. aureus, Aeromonas hydrophila*, and *E. coli* were cultured according to routine laboratory protocols. Entire experiments were performed following recommendations of the Laboratory Animals (Ministry of Science and Technology of China 2006) and approved by the Animal Ethics Committee of Zhongkai University of Agriculture and Engineering.

### Isolation of Purified Erythrocytes

The goose erythrocytes were isolated and purified according to a previously described procedure with minor modification (20). Briefly, blood samples were collected from a wing vein using heparinized syringes and mixed with 0.9% buffered saline (0.9 g NaCl in 100 ml H_2_O) containing heparin sodium (0.1 mg/ml). After washing with 0.9% buffered saline by centrifugation, the cell suspension was layered onto a 34%/51% Percoll (Pharmacia Fine Chemicals, Uppsala, Sweden) density gradient and centrifuged at 500 × *g* for 30 min at 4°C. After the plasma and white blood cells were removed (21), the RBC pellets were collected, washed three times, and finally resuspended in 0.9% buffered saline. The 5% trypan blue solution was used to make sure the cell viability >95%. The isolated erythrocytes were cultured in L15 medium containing 10% calf serum (Gibco, Carlsbad, USA), 100 U/mL penicillin (Gibco, Carlsbad, USA), 100 µg/mL streptomycin (Gibco, Carlsbad, USA), and 50 µg/mL gentamicin (Gibco, Carlsbad, USA).

### Fluorescent Bead Adhesion Assay

An aliquot of purified erythrocytes (1 × 10^6^cells/ml) was incubated with beads of different sizes (0.1 μm, 0.5 μm, 1.0 μm, and 2.0 μm, Sigma, USA) in a 1:10 ratio for 2 h at 37°C. Later, the suspensions were washed three times with sterile 0.9% buffered saline to remove non-adherent beads and observed under a fluorescence microscope (Leica DMI8, Germany). The adhesion rate was analyzed using a FACScan flow cytometer (BD Biosciences, USA). Next, another group of purified erythrocytes incubated with 0.8-μm beads (Sigma, USA) at the ratio of 1:10. The mixture was incubated for 1 h, 2 h, and 4 h at 37°C. After washing, bright-field photographs were obtained under a fluorescence microscope.

### Observation of Goose RBC Phagocytosis Morphology

The purified goose red blood cells (1 × 10^6^ cells/ml) were incubated with latex beads (0. 8-μm diameter) at a ratio of 1:10. After incubation at 37°C for 1 h, 2 h, and 4 h, the beads were washed three times with 0.9% sterile buffered saline and fixed in 2.5% glutaraldehyde at 4°C for at least 2 h. Finally, scanning electron microscopy (SEM) (Hitachi SU8010, Japan) and transmission electron microscopy (TEM) images (Hitachi H-7000FA, Japan) were used to observe the phagocytic activity of goose RBC. Scanning electron microscope magnification (× 8.00 k and × 20.0 k) and transmission electron microscopy magnification (× 4.0 k).

### Bacterial Adhesion Assay

Fluorescein isothiocyanate (FITC) was stained and labeled with *S. aureus*, *A. hydrophila* and *E. coli* at a ratio of 1:100, respectively, and incubated in a constant temperature incubator at 37°C for 30 min sheltered from light. After that, the labeled bacterial solution was resuspended to a concentration of 10^7^ CFU/ml by using 0.9% sterile buffered saline. Purified erythrocytes (1 × 10^6^ cells/ml) were incubated with fluorescein isothiocyanate (FITC)-labeled *S. aureus*, *A. hydrophila*, and *E. coli* (1 × 10^7^ CFU/ml) at a ratio of 1:10 in an incubator at 37°C for 1 h, 2 h, and 4 h. Later, the suspensions were washed three times with 0.9% sterile buffered saline to remove unattached bacteria, and the phagocytosis rate was analyzed by a FACScan flow cytometer (BD Biosciences, USA).

### Detection of RBC Reactive Oxygen Species (ROS) and Nitric Oxide (NO) Production Levels by Flow Cytometry

Experiments were performed according to the instructions of the kit, and levels of ROS and iNOS in erythrocytes were measured with the fluorescent probe 2’-7’-dichlorodihydrofluorescein-diacetate (DCFH-DA, Sigma, USA), which penetrates cells and hydrolyzes to non-fluorescent dichlorodihydrofluorescein (DCFH). NO production was measured by the fluorescence probe DAF-FM (Sigma, USA). Briefly, purified erythrocytes (1 × 10^5^ cells/ml) were incubated with *S. aureus* (10^6^ CFU/ml), *A. hydrophila* (10^6^ CFU/ml), and *E. coli* (10^6^ CFU/ml) at 1 h, 2 h, and 4 h, and suspensions were collected at these time points. The mixtures were incubated with 1 μmol/ml DCFH-DA (Sigma, USA) or DAF-FM (Sigma, USA) for 25 min at 37°C in a sheltered room, and then, the cells were washed three times with 0.9% sterile buffered saline and resuspended in 500 μL 0.9% sterile buffered saline for analysis with a FACScan flow cytometer (BD Biosciences, USA).

### RNA Extraction and cDNA Synthesis

The purified erythrocytes (1 × 10^6^ cells/ml) were incubated with *S. aureus* (10^7^ CFU/ml), *A. hydrophila* (10^7^ CFU/ml), and *E. coli* (10^7^ CFU/ml) in an incubator at 37°C for 1 h, 2 h, 4 h, and 8 h. Afterwards, total erythrocytes RNA were obtained with RNAiso Plus according to the manufacturer’s protocol. Approximately 1 µg of total RNA was used to generate cDNA. qRT-PCR was performed using HIScript^®^Q Select RT SuperMix (Vazyme, Nanjing, China) according to the manufacturer’s instructions, and then stored at -20°C. Primers were designed by Primer Premier 5.0, and listed in **Table 1**.

**Table 1:** List of primers used for RT-Qpcr.

Gene name	Forward primer (5’-3’)	Reverse primer (5’-3’)
β-actin	CGGCAACGAGCGGTTCAGGT	GGGTACATGGTGGTGCCGCC
IL-1β	CGACATCAACCAGAAGTGCTTC	ACGACATGTAGAGCTTGTAGCC
IL-8	GATGTGAAGCTGACGCAAAGTG	GTGCATCAGAATTGAGCTGAGC
TLR4	AGGGAAAGAGGAATGGTGTTG	CACCCAAGAGAAAGCACAAAG
NOX1	CATGGAGGTGGGACAGTACATC	TGGATGGAGAAGAAGTCCTCCT
NOX5	CGTTGAGCTGACATCGTACAGA	CGCTGTTTATAGTCGTGGCTCT

### qRT-PCR Assay

The qRT-PCR was performed in a qTOWER3 real-time PCR thermocycler (Analytik Jena AG, Germany), following the protocol for the AceQ qPCR SYBR Real-time PCR Premixture (Vazyme, Nanjing, China). In this study, two subunits of NADPH enzymes, NOX1 and NOX5, were selected for expression analysis, and β-actin was used as an internal reference gene. The qRT-PCR procedures were 5 min at 95°C, followed by 40 cycles consisting of 15 sec at 95°C, 30 sec at 60°C, and 20 sec at 72°C, and lastly, for 5 min at 4°C. There were three replicates for each treatment, and all technical experiments were triplicated (22). The 2^-ΔΔCT^ method was used to calculate the mRNA levels (23).

### The Measurement of Erythrocytes Antibacterial Activity

We further investigated the antimicrobial activity of erythrocytes, which was performed by the previously described method, with minor modifications (12). Briefly, each 1 ml of erythrocytes (5 × 10^6^ cells/ml) was incubated with *S. aureus* and *E. coli* at 37°C for 1 h, 2 h, 4 h, and 8 h, after which the infected cells were serially diluted 10-fold with sterile saline, spread on nutrient agar plates, and incubated overnight at 37°C. The antibacterial activity of the erythrocytes was determined by observing how the number of bacterial colonies on the agar plates differed from the control group.

### The Measurement of Erythrocytes Antioxidant Enzyme Activity

The activities of superoxide dismutase (SOD), catalase (CAT), and glutathione peroxidase (GSH-Px) were measured independently in the purified goose erythrocytes (5 × 10^6^ cells/ml) after treated with *S. aureus*, *A. hydrophila*, and *E. coli* for 1, 2, and 4 h. Bacterial concentration adjusted to (5×10^7^ CFU/ml) at 10-fold dilution. SOD, CAT, and GSH-Px assay kits (Nanjing Jiancheng Bioengineering Institute, Nanjing, Jiangsu, China) were used to determine the response of antioxidant system in goose erythrocytes SOD, CAT, and GSH-Px according to the manufacturer’s instructions.

### Statistical Analysis

The data were statistically analyzed using SPSS software (version 23.0). All data are expressed as the mean ± SEM. Statistical significance was measured by one-way analysis of variance (ANOVA) and t-test, using graphs drawn with GraphPad Prism 7 software. The differences were statistically significant at P < 0.05 (*) or P < 0.01 (**).

## Results

### Phagocytosis of Latex Beads of Different Diameters by Goose Erythrocytes

**Figure 1:**
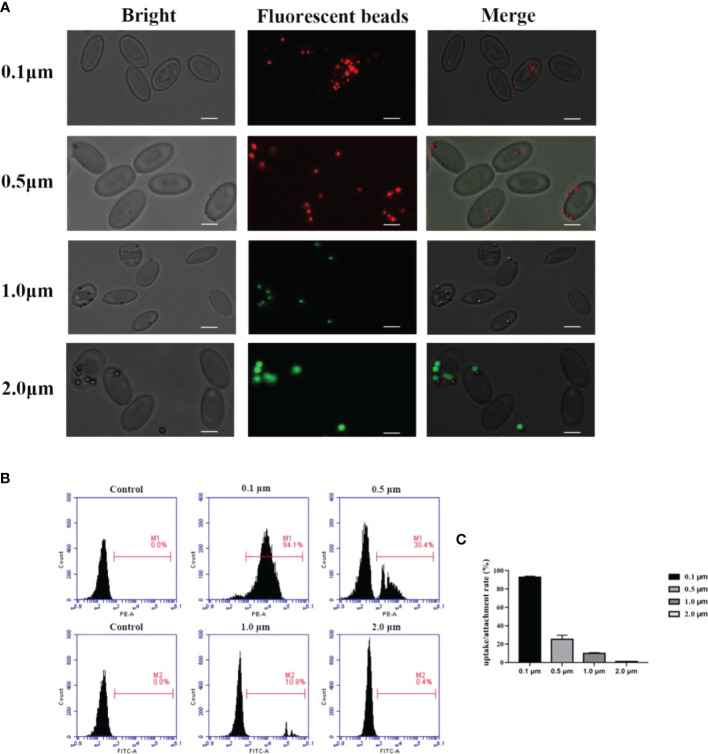
Observation of goose red blood cells associate with beads of different sizes by fluorescence microscopy. **(A)** Erythrocytes were co-incubated with 0.1 μm, 0.5 μm, 1.0 μm and 2.0 μm latex beads (0.1 μm and 0.5 μm labelled red, 1.0 μm and 2.0 μm labelled green). The ruler represents 5 μm. Efficiency of goose Erythrocytes in phagocytosis of latex beads of different sizes. **(B)** Erythrocytes were incubated with microspheres of different sizes (0.1 μm, 0.5 μm, 1.0 μm, 2. 0 μm) for 2h, the phagocytosis rate of erythrocytes was measured by flow cytometry. Results are representative of three experiments. **(C)** Rate of association of erythrocytes with latex beads of different sizes. Data are the mean values of three independent experiments.

The fluorescence microscopy was used to detect the ability of the goose erythrocytes to uptake and attach different size of beads (0.1 μm, 0.5 μm, and 1.0 μm). As shown in **Figure 1A**, numerous fluorescence of different size of beads were recorded over the surface or inside of the individual erythrocytes. Followed, flow cytometric analysis showed that goose erythrocytes were able to uptake different diameters of fluorescent latex beads with different efficiencies (**Figures 1B, C**). Specifically, the erythrocyte phagocytosis rates of 0.1 μm, 0.5 μm, 1.0 μm, and 2.0 μm microbeads were 94.1%, 30.4%, 10.9%, and 0.4%, respectively. After incubated with different sizes of fluorescent beads for 2 h, it was observed that 0.1 μm beads were more easily phagocytosed, followed by 0.5 μm beads. For 2.0 μm beads, the goose erythrocytes phagocytosis rate was much lower compared to the smaller beads (0.1 μm, 0.5 μm, and 1.0 μm) (**Figures 1B, C**).

### Phagocytic Activity of Goose Erythrocytes

**Figure 2:**
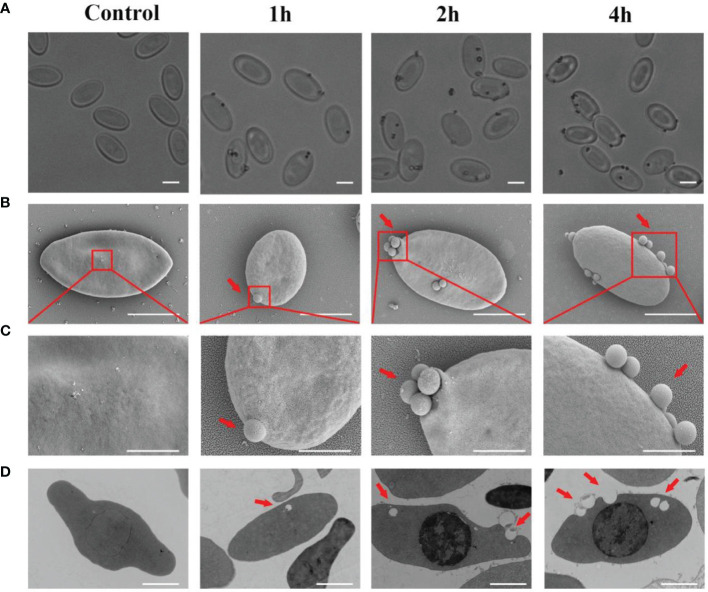
The attachment of 0. 8 μm latex beads with the cell surface of Erythrocytes. **(A)** Optical microscopic observation of erythrocyte phagocytosed latex beads. The ruler represents 5 μm. **(B)** Scanning electron micrographs was used to detect the adhesion of latex beads to goose Erythrocytes. SEM magnification (× 8.00 k). The ruler represents 5 μm. **(C)** The partial magnification of the red box in **Figure 3B**. SEM magnification (× 20.0 k). The ruler represents 2 μm. **(D)** Transmission electron microscopic observation of goose erythrocytes phagocytosing latex beads. TEM magnification (× 4.0 k). The ruler represents 2 μm.

To further test the phagocytic ability of goose erythrocytes, 0.8 μm latex beads were incubated with erythrocytes at 37°C for 1 h, 2 h, and 4 h. Under the optical microscopy, the 0.8 μm latex beads were found to be surrounded the goose erythrocytes (**Figure 2A**). Furthermore, SEM observation data showed that beads had tightly bound to erythrocytes and formed distinct convex vesicle structures over the surface of the RBC (**Figure 2B**). The SEM analysis was performed to characterize the phagocytosis of goose erythrocytes, and the results showed that some 0.8 μm latex spheres attached to the surface of individual erythrocytes and then formed invaginations, which were partially phagocytosed (**Figure 2C**). To further confirm the phagocytic activity of erythrocytes, which were observed under TEM, and it was clear that latex beads were also visible within the erythrocytes (**Figure 2D**).

### Phagocytosis and Adhesion Activity of Goose Erythrocytes Against Bacteria

**Figure 3:**
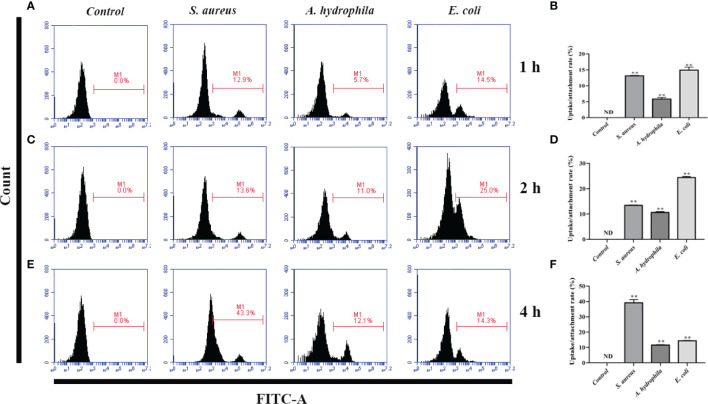
Flow cytometric assay of phagocytic activity of goose erythrocytes against bacteria. The goose erythrocytes were co-incubated with FITC-labeled *S. aureus*, *A. hydrophila* and *E. coli* for 1 h, 2 h and 4 h. 1 h uptake/attachment rate **(A, B)**, 2 h uptake/attachment rate **(C, D)** and 4h uptake/attachment rate **(E, F)** was measured by flow cytometry. **P < 0. 01. Data represent the mean ± SEM of three independent experiments. ND, not detected.

To investigate the phagocytosis and adhesion ability of goose erythrocytes towards bacteria, we incubated goose erythrocytes with *S. aureus*, *A. hydrophila*, and *E. coli* for 1 h, 2 h, and 4 h. By flow cytometry analysis, the phagocytosis and adhesion activity of *E. coli* by erythrocytes was increased from 1 h to 4 h (**Figures 3B, D**), but decreased at 4 h (**Figure 3F**), and the phagocytosis and adhesion activity for *S. aureus* by goose erythrocytes increased with time (**Figures 3A, C, E**). For *A. hydrophila*, the phagocytosis and adhesion rate at 2 h and 4 h were significantly higher than that of in 1 h (**Figures 3C, E**).

### Bacteria-Induced ROS/iNOS Production in Erythrocytes

**Figure 4:**
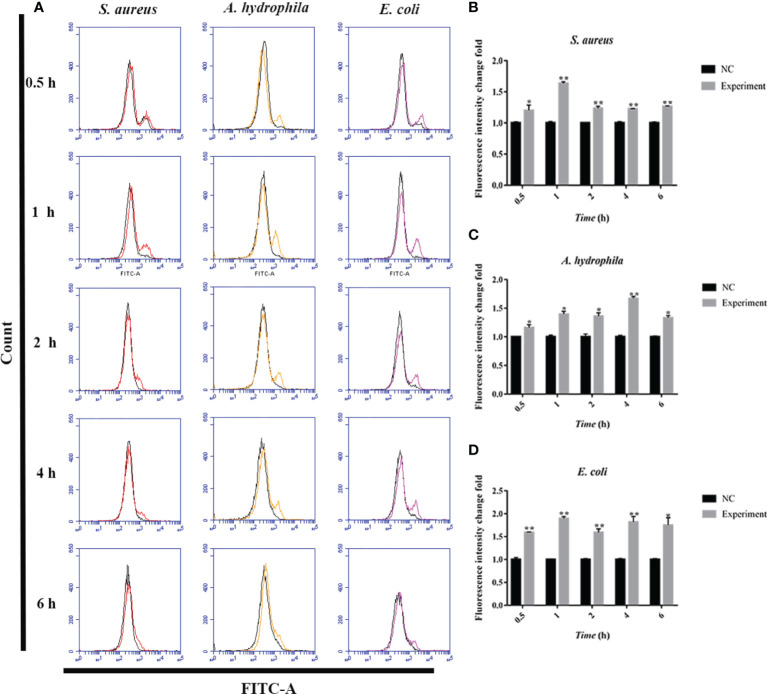
Bacterial stimulation of erythrocytes to produce ROS. **(A)**A flow cytometric assay is performed to detect ROS produced by erythrocytes co-incubated with three bacteria (*S. aureus, A. hydrophila and E. coli*) for 0.5 h, 1h, 2 h, 4 h and 6h, respectively. **(B)** ROS production by flow cytometry in *S. aureus* stimulated goose Erythrocytes. **(C)** ROS production by flow cytometry in *A. hydrophila* stimulated goose Erythrocytes. **(D)** ROS production by flow cytometry in *E. coli* stimulated goose Erythrocytes. *P < 0. 05; **P < 0. 01. Data represent the mean ± SEM of three independent experiments.

**Figure 5:**
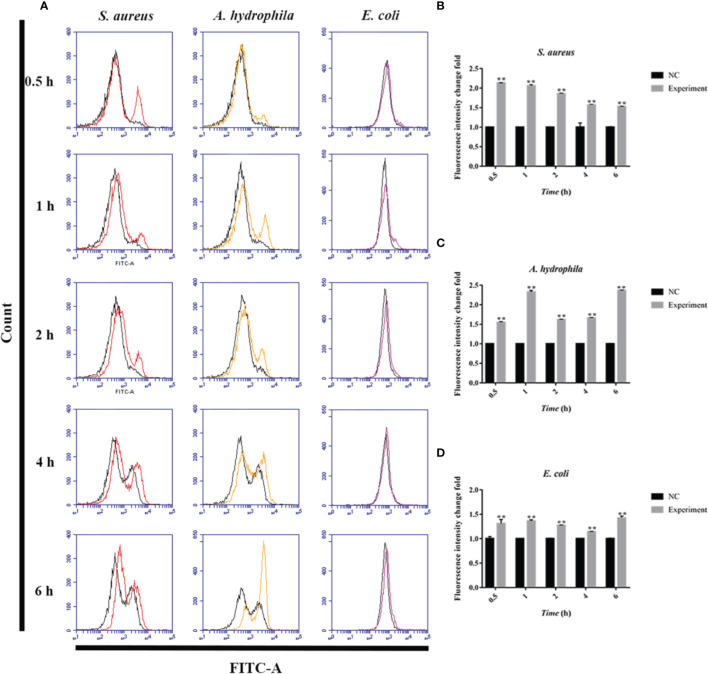
Bacterial stimulation of erythrocytes to produce iNOS. **(A)** A flow cytometric assay is performed to detect ROS produced by erythrocytes co-incubated with three bacteria (*S. aureus, A. hydrophila and E. coli*) for 0.5 h, 1h, 2 h, 4 h and 6h, respectively. **(B)** iNOS production by flow cytometry in *S. aureus* stimulated goose erythrocytes. **(C)** iNOS production by flow cytometry in *A. hydrophila* stimulated goose erythrocytes. **(D)** iNOS production by flow cytometry in *E. coli* stimulated goose erythrocytes **(D)**. **P < 0. 01. Data represent the mean ± SEM of three independent experiments.

To further investigate the antimicrobial mechanism of goose erythrocytes, we measured the ROS and iNOS levels of erythrocytes stimulated by three kinds of bacteria for 0.5, 1, 2, 4 and 6 h. The flow cytometry analysis data revealed that the stimulation of *S. aureus* significantly increased the ROS production compared to control groups, and reached the highest level at 1 h (**Figures 4A, B**). In *A. hydrophila* stimulation groups, the production of ROS has a highest level at 4 h, and the production of ROS in *A. hydrophila* stimulation groups were significantly higher than that in control groups (**Figures 4A, C**). Similar to *S. aureus* stimulation groups, the stimulation of *E. coli* significantly increased the production of ROS at all tested time points, and at 1 h showed a highest level (**Figures 4A, D**). In determination of iNOS assay, the results data showed that the stimulation of *S. aureus* significantly increased the iNOS production at all tested time points, and at 0.5 h showed the highest iNOS levels (**Figures 5A, B**). In *A. hydrophila* and *E. coli* stimulation groups, the data showed that the highest iNOS level both at 6 h, and the production of iNOS at all time points were significantly higher than that of in control groups (**Figures 5A, C, D**).

### The Regulation of ROS in Goose Erythrocytes From NADPH Enzymes

**Figure 6:**
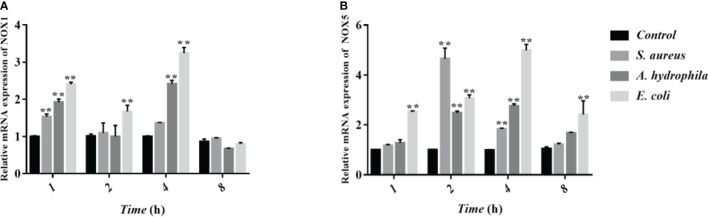
Determination of transcript levels of erythrocyte NADPH enzymes by qRT-PCR. **(A, B)** Detection of NADPH subunit NOX1 and NOX5 transcript levels by qRT-PCR. These data are expressed as the mean ± SEM relative to the control. **p < 0. 01 vs. control.

NOX family NADPH oxidases are proteins that transfer electrons across biological membranes. Usually, the electron acceptor is oxygen and the product of the electron transfer reaction is superoxide. Therefore, the biological function of NOX enzymes is to generate ROS. The homologs of the catalytic subunit of NADPH oxidase, namely NOX1, NOX2, NOX3, NOX4, NOX5, DUOX1, and DUOX2 which are all involved in the regulation of ROS in macrophages. To further verify whether NADPH in erythrocytes is involved in the regulation of ROS, we measured the transcript levels of two subunits of NADPH enzymes, NOX1 and NOX5 through qRT-PCR. The results showed that after stimulation by *S. aureus*, *A. hydrophila*, and *E. coli*, NOX1 transcript levels were significantly upregulated at 1 h and 4 h with a significant difference (P < 0.01), and NOX5 transcript levels were significantly upregulated at 2 h and 4 h with a significant difference (P < 0.01) (**Figure 6**).

### The Antimicrobial Activity of Goose Erythrocytes

**Figure 7:**
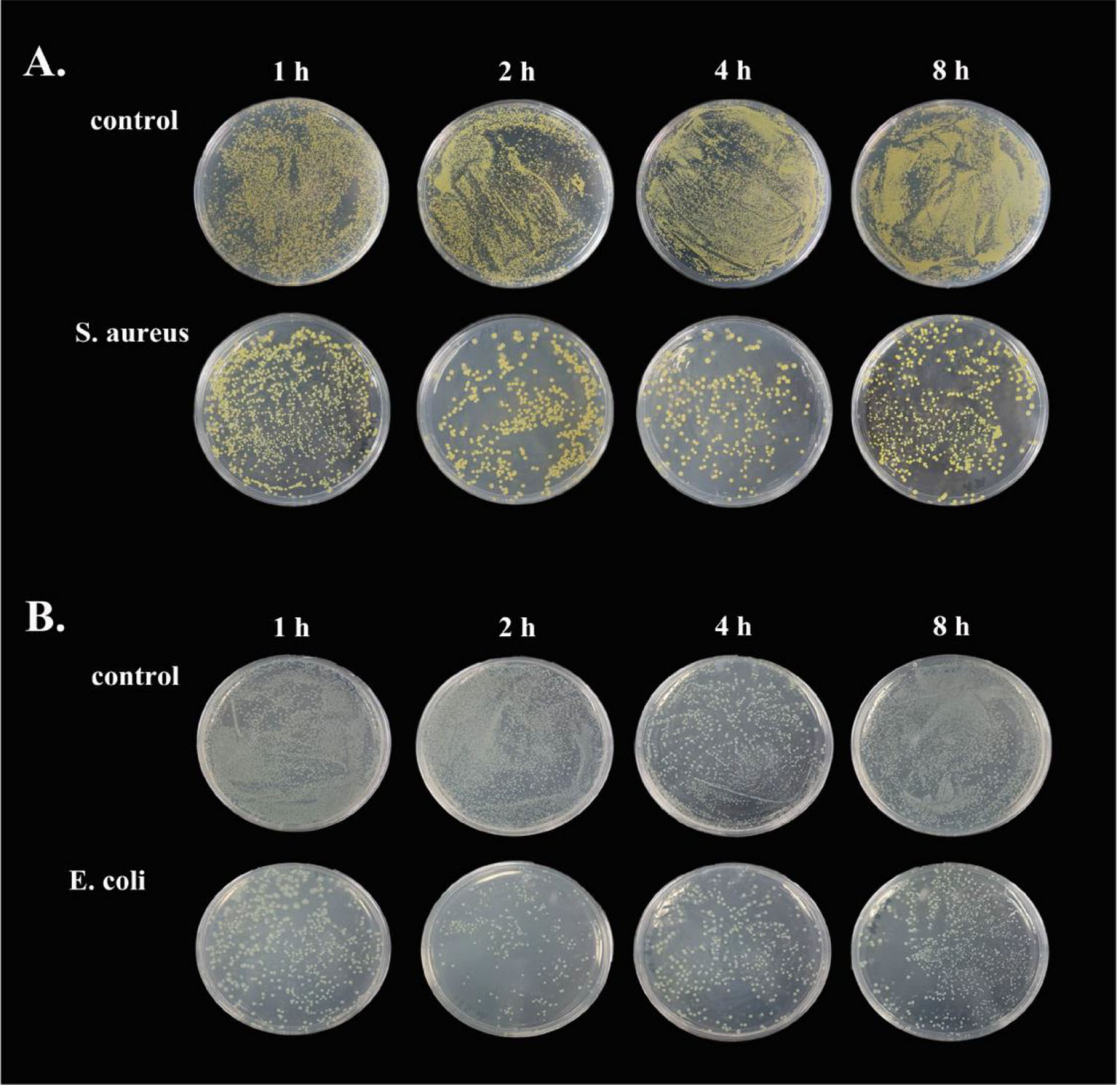
The antibacterial ability of goose Erythrocytes. **(A)** Goose Erythrocytes were incubated with *S. aureus* for 1 h, 2 h, 6 h and 8 h after coating the plates for observation. **(B)** Goose Erythrocytes were incubated with *E. coli* for 1 h, 2 h, 6 h and 8 h after coating the plates for observation.

To demonstrate the antimicrobial ability of goose erythrocytes, we performed antibacterial experiments with erythrocytes acting against *S. aureus* and *E. coli.* The results showed that the erythrocytes exhibited strong activity against both bacteria, with erythrocytes showing stronger antibacterial activity against *S. aureus* at 2 h and 4 h compared to the control (**Figure 7A**), and against *E. coli* at 2 h and 4 h (**Figure 7B**).

### The Incubation of Bacteria Activated the Antioxidant Enzyme System in Goose Erythrocytes

**Figure 8:**
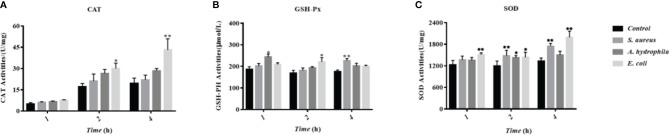
The influence of bacterial infestation on the activity of antioxidant enzymes in goose erythrocytes. The CAT **(A)**, GSH-Px **(B)** and SOD **(C)** of erythrocyte are measured after co-incubation of erythrocytes with three bacteria (*S. aureus*, *A. hydrophila* and *E. coli*) for 1h, 2h and 4h. * p < 0. 05 or ** p < 0. 01 vs. control. Data represent the mean ± SEM of three independent experiments.

To investigate the influence of bacterial incubation to the antioxidant system in goose erythrocytes, the antioxidant indicators including CAT, GSH-Px, SOD, and other antioxidant enzyme activities were measured, whose basic function is to scavenge superoxide radicals such as superoxide anion (O^2-^) and hydrogen peroxide (H_2_O_2_). The results showed that the incubation of three bacteria resulted in an increase in the CAT activity with time, especially a significant increase at 2 h and 4 h with significant differences (P < 0.01) (**Figure 8A**). All three bacteria stimulated GSH-Px activity, which increased at 1 h, 2 h, and 4 h, with a significant increase by *S. aureus* at 4 h with significant differences (P < 0.01) (**Figure 8B**). A significant increase in SOD activity was also observed at 2 h and 4 h with significant differences (P < 0.01) (**Figure 8C**).

### Inflammatory Response to Bacterial Stimulation in Goose Erythrocytes

**Figure 9:**
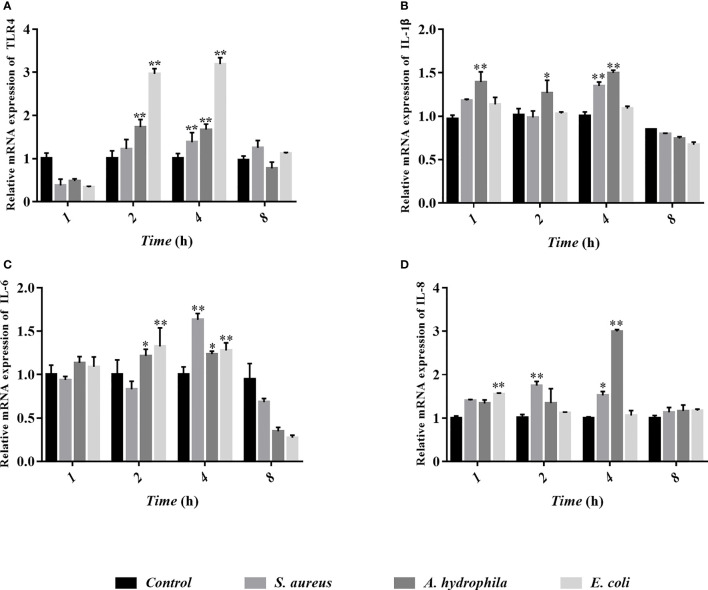
Analyses of erythrocytes immune-related gene expression with qRT-PCR after co-incubation of erythrocytes with three bacteria (*S. aureus, A. hydrophila* and *E. coli*) for 1h, 2h, 4h and 8h. The detected genes including TLR4 **(A)**, IL-1β **(B)**, IL-6 **(C)**, and IL-8 **(D)**, and these data are expressed as the mean± SEM relative to the con-trol. *p < 0. 05 vs. control; **p < 0. 01 vs. control.

To study the behavioral characteristics of goose erythrocytes during bacterial infection, TLR4, IL-1β, IL-6, and IL-8 were measured by qRT-PCR after *S. aureus*, *A. hydrophila*, and *E. coli* stimulation. We found that TLR4 gene expression was significantly upregulated in goose erythrocytes after 2 to 4 h of stimulation by the three bacteria (**Figure 9A**), but there was no significant difference (P > 0.05) in *S. aureus* stimulation at 2 h (**Figure 9B**). The expression of the IL-1β gene was significantly upregulated after *A. hydrophila* stimulation for 1 h, while the expression of the IL-1β gene was significantly upregulated after *S. aureus* and *A. hydrophila* stimulation for 4 h, with significant differences (P < 0.01). However, the expression of the IL-1β gene was upregulated but was not significantly different (P < 0.01) after *E. coli* stimulation from 1 to 4 h (**Figure 9B**). In addition, IL-6 gene expression levels were significantly upregulated during *E. coli* stimulation from 2 to 4 h with significant difference (P < 0.01), while for *S. aureus* stimulation at 4 h, IL-6 gene expression levels were significantly upregulated with significant difference (P < 0.01). In contrast, IL-6 gene expression levels were upregulated during *A. hydrophila* stimulation for 2 to 4 h, but were not significantly different (P < 0.01) (**Figure 9C**). IL-8 gene expression levels were significantly upregulated during *E. coli* stimulation for 1 h, while after *S. aureus* and *A. hydrophila* stimulation for 2 to 4 h, IL-8 gene expression levels were significantly upregulated with a significant difference (P < 0.01) (**Figure 9D**).

## Discussion

Phagocytosis was discovered in 1882 (24, 25), and it is an important key process performed by phagocytes that removes pathogens and cellular debris. The ability to phagocytose large foreign bodies or invasive microorganisms can protect the organism from foreign pathogenic microorganisms and harmful particulate matter. Phagocytes are classified as specialized phagocytes (macrophages, neutrophils, and dendritic cells) and non-professional phagocytes (fibroblasts and endothelial cells). Phagocytes are the important component in the natural immune response and are the primary line of defense of the host against invading pathogens. Interestingly, Nelson observed in 1953 that human erythrocytes were involved in the natural immune response of the organism and had an immune adhesion role (7), which suggests that erythrocytes may also have a function similar to that of phagocytes.

It is well known that the major function of erythrocytes is to deliver oxygen to the tissues of the body (26). Modern studies have shown that erythrocytes also participate in immune regulation and immunologically relevant functions. According to previous research reports, senescent erythrocytes can be phagocytosed in the spleen through a non-inflammatory pathway (27). In addition, erythrocytes also regulate T cell proliferation, and promote the secretion of related cytokines (28, 29). Erythrocytes capture immune complexes and bacteria and deliver them to professional antigen-presenting cells (APCs) in the spleen, whereas carrier erythrocytes are not captured (30–32). Therefore, in addition to the leukocytes in the blood, erythrocytes also play a bactericidal role in human blood and are an indispensable component of the body’s immune system (26). However, the phagocytic properties and antimicrobial capacity of avian erythrocytes are not yet known.

In this study, we have reported the potential mechanism and antimicrobial activity of avian erythrocytes. Fluorescence microscopy showed that there was comparable phagocytosis of latex beads with diameters of 0.1 μm, 0.5 μm, 1.0 μm, and 2.0 μm by goose erythrocytes. According to the flow cytometry results, there was relatively high phagocytosis of latex beads with diameters of 0.1 μm, 0.5 μm, and 1.0 μm, while the phagocytosis of 2.0-μm diameter latex beads was significantly reduced to only approximately 0.4%. These results indicate that goose erythrocytes participated in relatively stable phagocytosis of particles in the size range of 0.1-1.0 μm. The SEM and TEM observation results showed that goose erythrocytes captured additional latex beads over time. According to recent studies, researchers have observed phagocytic capacity of B cells in many species of fish, such as rainbow trout *(Oncorhynchus mykiss L.)*, *Atlantic salmon (Salmo salar L.)*, Atlantic cod *(Gadus morhua L.)*, lumpfish *(Cyclopterus lumpus L.)*, Japanese flounder *(Paralichthys olivaceus)*, and turbot *(Scophthalmus maximus)* (33–38). The similar results also be reported in erythrocytes of grass carp (16). The phagocytosis efficiency is likely to be related to the choice of particles (latex beads, bacteria, or spores) to be phagocytosed, the shape of the particles, the incubation temperature and time, share the similar to that of lymphocytes and macrophages (39–41). Therefore, we examined the phagocytic activity of goose erythrocytes using three kinds of bacteria, and the flow cytometry results showed that goose erythrocytes possess extensive phagocytic activity against microorganisms. These findings were similar to those that erythrocytes of rainbow trout can bind and phagocytose *Candida albicans* (15).

The microbicidal mechanisms of macrophages comprise of the activation of NADPH oxidase and inducible nitric oxide synthase (iNOS) in facilitating the ROS and RNS synthesis, respectively (42). Here, flow cytometry was used to verify whether the goose erythrocytes can produce ROS and iNOS after incubated with of *S. aureus*, *A. hydrophila*, and *E. coli*, similar to those of reported experiments in which macrophages killed Salmonella (43), where the sterilization mechanism is likely to be immune clearance through the killing effect of ROS and RNS (44). NADPH oxidases consists of several NOX subunits (NOX1-5, DUOX1, DUOX2), while they are the main source of ROS in non-phagocytic cells (45, 46). As early as 1964, Rossi and Zatti accurately proposed that NADPH oxidase was the cause of respiratory burst (47). In this study, NOX1 and NOX5 were selected to determine the response of the NADPH oxidase (NOX) family after stimulated by three kinds of bacteria. The results of this experiment verified that the expression level of NOX1 and NOX5 were significantly up-regulated, which was consistent with the ROS production determination. We also explored the bacterial growth of *S. aureus* and *E. coli* after co-incubation with goose erythrocytes for a period of time, by means of coated plates. The number of colonies were visually observed a significant decreased in goose erythrocytes groups, which further indicated that ROS might be involved in the antibacterial activity.

A complex antioxidant defense system composed of non-enzyme glutathione GSH and antioxidant enzymes (SOD, CAT, and GSH-Px) can effectively scavenge ROS (48). In poultry rearing, antioxidant enzymes are used as the first line of defense of the organism against oxidative stress, and they are beneficial in the prevention of oxidative damage (49–51). In this study, the data was shown that the upregulation of antioxidant stress levels in goose erythrocytes is triggered after bacterial stress. This is most likely due to the fact that goose erythrocytes produce excess ROS to kill bacteria, thus triggering the antioxidant defense system to prevent the excess ROS from causing oxidative damage to the organism. Moreover, a complex series of inflammatory responses result when many harmful stimuli, such as pathogens and bacteria. The onset of inflammation is triggered by interactions between cell surface toll-like receptors, such as TLR-4 and TLR-2, along with their ligands (52). Inflammatory biomarkers, such as interleukin-1β (IL-1β), interleukin-6 (IL-6), and interleukin-8 (IL-8), can mediate the inflammatory and immune response of the organism when cells are infected with microorganisms or stressed, thus enabling them to kill intracellular pathogens (53). In the present study, the stimulation of three different bacteria significantly increased the expression of TLR-4, pro-inflammatory cytokines IL-1β, IL-6, and IL-8.

In summary, in this study, we clearly revealed the immune adhesion and phagocytosis of avian erythrocytes against pathogenic bacteria. We found that goose erythrocytes were involved in immune regulation of the organism and possessed some antimicrobial activity, while killing bacteria by producing potent ROS. These findings provide evidence for the involvement of avian erythrocytes in the innate immunity of the organism, and provide new insight into the mechanism of action that governs the antimicrobial activity of avian erythrocytes.

## Data Availability Statement

The raw data supporting the conclusions of this article will be made available by the authors, without undue reservation.

## Ethics Statement

Entire experiments were performed following recommendations of the Laboratory Animals (Ministry of Science and Technology of China 2006) and approved by the Animal Ethics Committee of Zhongkai University of Agriculture and Engineering.

## Author Contributions

YY performed experiments, analyzed data, and wrote the manuscript. JC, LLu, ZX, FL, and MY performed experiments. LLi and ZQ conceived ideas, analyzed data, oversaw the research, and wrote the manuscript. JL contributed to the preparation of the manuscript. All authors contributed to the article and approved the submitted version.

## Conflict of Interest

The authors declare that the research was conducted in the absence of any commercial or financial relationships that could be construed as a potential conflict of interest.

## Publisher’s Note

All claims expressed in this article are solely those of the authors and do not necessarily represent those of their affiliated organizations, or those of the publisher, the editors and the reviewers. Any product that may be evaluated in this article, or claim that may be made by its manufacturer, is not guaranteed or endorsed by the publisher.
